# Supplementary material for: Sugar restriction and blood ingestion shape divergent immune defense trajectories in the mosquito Aedes aegypti
Source: Sci Rep. 2023 Jul 31;13:12368. doi: 10.1038/s41598-023-39067-9 (PMC10390476; doi:10.1038/s41598-023-39067-9)
Supplement: Supplementary file 3 — Supplementary Information 3. [file 41598_2023_39067_MOESM3_ESM.pdf]

Supplementary Information

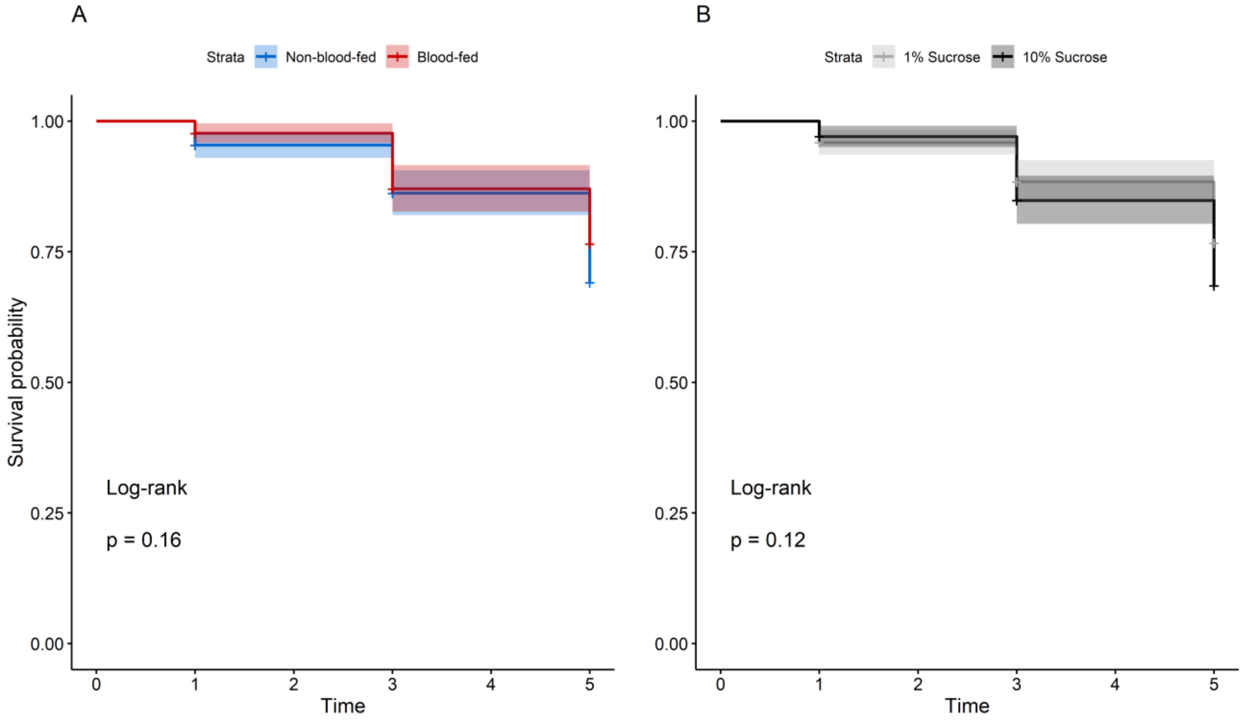

S1 Fig. Survival across a 5-day infection timecourse is affected by neither blood nor sucrose. Kaplan-Meier curves are displayed to compare the effects of (A) blood ingestion and (B) dietary sucrose concentration on survival in mosquitoes infected with *E. coli*. Corresponding log-rank test p-values are displayed on each panel.

S1 File. Selected R code used for statistical analysis and figures.

S1 Dataset. Data used in analyses. Data are divided into three tabs: final dataset (df_final), survival analysis dataset (df_surv), and raw CFU dilution counts (raw CFU counts) used to calculate medians used in df_final.
